# Regulatory Effect of Resveratrol and Prednisolone on MDR1 Protein Expression in Acute Lymphoblastic Leukemia Cell Line (CCRF-CEM)

**DOI:** 10.31557/APJCP.2019.20.4.1171

**Published:** 2019

**Authors:** Mehdi Talebi, Sina Bahar Aghdam, Ako Azimi, Hamed Mohammadi, Somayyeh Karimi Yonjali, Maryam Asariha, Milad Zadi Heydarabad

**Affiliations:** 1 *Hematology and Oncology Research Center,*; 2 *Immunology Research Center,*; 4 *Stem Cell Research Center, Tabriz University of Medical Sciences, Tabriz,*; 3 *Department of Basic Sciences, Maragheh University of Medical Sciences, Maragheh,*; 5 *Medical Biology Research Center, *; 6 *Student Research Committee, Kermanshah University of Medical Sciences, Kermanshah,*; 7 *Medicinal Plants Research Center, Yasuj University of Medical sciences, Yasuj, Iran. *

**Keywords:** Protein expression, acute lymphoblastic leukemia, resveratrol, prednisolone, MDR1 gene

## Abstract

**Objective:**

Chemotherapy is the most widely recognized technique to regard leukemia and also different sorts of human tumors. In any case, tranquilize protection has stayed as the primary test against the adequacy of medications. Besides, having different unfriendly impacts, chemotherapy drugs are getting to be traded by characteristic modalities for growth treatment. In such manner, natural segments, for example, resveratrol and prednisolone have been recognized to sharpen the leukemic cells to modified cell demise through an arrangement of complex procedures. In this investigation, we have analyzed effect of 15, 50 and 100μM of resveratrol and 700μM of prednisolone on the human multidrug protection quality 1 (MDR1) as a notable marker for cell sedate protection. We assessed the impact of resveratrol and prednisolone on MDR1 protein expression in the CCRF-CEM cell line as an agent for intense lymphoblastic leukemia. The investigation was planned to clear up whether.

**Materials and methods:**

CCRF-CEM cells linage get under drug treatment with use of resveratrol and prednisolone. Western blot use at 24 and 48 hours with different doses of resveratrol and prednisolone to analysis of MDR1 expression changes.

**Results:**

Effect of 15, 50, and 100 micro molar of resveratrol and 700 micro molars of prednisolone on CCRF-CEM cells led to the MDR1 decrease. Western blot use for evaluation of MDR1 protein expression changes.

**Conclusion:**

In the present study, we observed that resveratrol and prednisolone, with a dose-dependent effect, can reduce the expression of the MDR1 protein. This reduction of expression demonstrates that resveratrol and prednisolone can overcome to drug resistance created by MDR1.

## Introduction

An obtrusive and quickly developing growth of White Blood Cells (WBC) is the Acute Lymphoblastic Leukemia (ALL) which generally starts from bone marrow (Azimi et al., 2015; Azimi et al., 2016; Ahani-Nahayati et al., 2018). ALL is presently considered as the most common threat in youngsters, containing 77% of leukemic cases in this age gathering which is most every now and again happened in offspring of 2-5 years of age (Stanulla and Schrappe, 2009; Azimi et al., 2015; Azimi et al., 2016; Zadi Heydarabad et al., 2018). Since the revelation of ALL, few lines of medication specialists have centered to create compelling and safe modalities to keep the development and movement of this threat. An extensive variety of cures were planned with different sorts of systems; however, their danger and antagonistic impacts have been reliably remained the principle hindrance in front of productive treatment. Concerns about the danger and wide range of reactions, these days expanding interests are concentrated to supplant the concoction specialists by more regular or plant-based parts to treat the human diseases (Zhang et al., 2010; Rauf et al., 2017). 

**Table 1:** Women’s Sociodemographic Characteristics (n=255)

Characteristics	n (%)
Women’s age group	
16-20	9 (3.5)
21-25	39 (15.3)
26-30	75 (29.4)
31-35	57 (22.4)
Above 35	76 (29.4)
Mean:32	
Women’s level of education	
Never went to school	27 (10.7%)
Basic schooling	41 (16.1)
Secondary Schooling	92 (36.1)
Diploma	13 (5.1)
University	82 (32.2)
Husband Educational level	
Never went to school	30 (11.8)
Basic schooling	26 (10.2)
Secondary Schooling	133 (52.2)
Diploma	10 (3.9)
University	49 (15.3)
Women’s professional career	
None	160 (62.7)
Health	7 (2.7)
Admin	19 (7.5)
Profession	20 (7.8)
Academic	49 (19.2)
Husband professional career	
None	188 (73.7)
Health	10 (3.9)
Admin	13 (5.1)
Profession	23 (9.0)
Academic	21 (8.2)
Women’s working stats	
Yes	38 (14.9)
No	217 (85.1)

**Table 2:** Marital, Obstetric and Gynecologic History of Study Population (n=255)

Characteristic	n (%)
Marriage years	
Single	5 (2.0)
Less than 10 year	129 (50.6)
10 years and more	121 (47.5)
	median=10.72 years
No. of pregnancies	
0	18 (7.1)
1-3	120 (47.1)
4-6	74 (29.0)
More than 6	43 (16.9)
	median=3.89
No. of deliveries	
0	51 (20.0)
1-3	124 (48.6)
4-6	55 (21.6)
More than 6	25 (9.8)
	median=2.95
No. of living children	
0	55 (21.6)
1-3	123 (48.2)
4-6	53 (20.8)
More than 6	24 (9.11)
	median=2.80
Ever used family planning methods	
Yes	133 (52.2)
No	122 (47.8)
Ever conduct comprehensive reproductive health assessment	
Yes	58 (22.7)
No	197 (77.3)

**Table 3:** Women’s Knowledge, Attitudes and Practices of Cervical Cancer (n=255)

Characteristics	n(%)
Ever heard of cervical cancer	
Yes	169 (66.3)
No	86 (33.7)
Sources of information	
Family/friends	22 (13.0)
Family doctor/GP	2 (1.2)
OBGYN doctor	11 (6.5)
Nurse	1 (0.6)
Media	133 (78.7)
Knowledge about risk factors for cervical cancer	
1.Early marriage	
Yes	49 (19.2)
no	50 (19.6)
Don’t know	156 (61.2)
2.Marrying more than one husband during reproductive age	
Yes	47 (18.4)
No	32 (12.5)
Don’t know	176 (69.1)
3.Close multiple pregnancies	
Yes	49 (19.2)
No	50 (19.6)
Don’t know	156 (61.2)
4.Smoking	
Yes	98 (38.4)
No	22 (8.6)
Don’t know	135 (52.9)
5.Nutritional habits	
Yes	64 (25.1)
No	46 (18.0)
Don’t know	145 (56.9)
6.Too many children	
Yes	44 (17.3)
No	62 (24.3)
Don’t know	149 (58.4)
7.Poor hygiene	
Yes	108 (42.4)
No	17 (6.7)
Don’t know	130 (51.0)
8.Low socioeconomic level	
Yes	46 (18.0)
No	50 (19.6)
Don’t know	159 (62.4)
9.Vaginal inflammation	
Yes	134 (52.5)
No	3 (1.2)
Don’t know	118 (46.3)
Number of risk factors recognized	
0	87 (34.1)
1-2	59 (23.1)
3-4	58 (22.7)
> 4	51 (20.0)
Direct cause of the cervical cancer	
HIV	1 (0.4)
HPV	0 (0.0)
Chlamydia	1 (0.4)
Don’t know	253 (99.2)
Cervical cancer can be prevented	
Yes	142 (55.7)
No	5 (2.0)
Don’t know	108 (42.4)
Ever Heard of vaccine for cervical Cancer (HPV)	
Yes	10 (3.9)
No	7 (2.7)
Don’t know	238 (93.3)
Have you ever took the vaccine	
Yes	1 (0.4 )
No	254 (99.6)

**Table 4:** Women’s Knowledge, Attitudes and Practices of Pap Smear Test (n=255)

Characteristics	n(%)
Have you heard of pap smear test	
Yes	111 (43.5)
No	144 (56.5)
Sources of information	
Family/friends	8 (7.2)
Family doctor/GP	2 (1.8)
OBGYN doctor	31 (27.9)
Nurse	3 (2.7)
Media	67 (60.4)
Pap smear can identify early asymptomatic lesions	
Yes	93 (36.5)
No	8 (3.1)
Don’t know	154 (60.4)
Early detection of cervical cancer has good effect on outcome of treatment	
Yes	142 (55.7)
No	5 (2.0)
Don’t know	108 (42.4)
If properly informed about Pap smear would you do it?	
Yes	99 (38.3)
No	76 (29.8)
Don’t know	80 (31.4)
If refuses to do Pap Smear, what are the causes?	
May be painful	11 (7.1)
I feel shy	7 (4.5)
I am healthy, no need	71 (45.5)
Husband would not agree	4 (2.6)
Physician doesn’t request	23 (14.7)
Don not know where to go	40 (25.6)
If you agree to do Pap Smear, whom do u prefer to do it?	
Family doctor	5 (5.1)
OBGYN	73 (73.7)
Private doctor	6 (6.1)
Mother and child health doctor	2 (2.0)
nurse	13 (13.1)
If you agree to do Pap smear, what is the preferred health provider gender?	
Male	5 (5.1)
Female	94 (94.9)
If you agree to do Pap smear, where is the preferred place?	
Women clinic in hospital	23 (23.1)
Obs. and Gyne in hospital	65 (65.7)
Private clinic	11 (11.1)

**Table 5:** The Relationship between Women’s Selected Characteristics and Women’s Knowledge, Attitudes, and Practices of Cervical Cancer and Cervical Cancer Prevention (n=255)

Characteristics (n)	Knowledge CCa	Knowledge Pap Smear	When properly informed about Pap smear, women would do it.	CC can be prevented
Women’s level of education				
Never went to school (27)	9 (33.3)	7 (25.9)	9 (33.3)	6 (22.2)
Basic schooling (41)	23 (56.1)	15 (36.6)	10 (24.4)	19 (46.3)
Secondary schooling (92)	64 (69.6)	39 (42.2)	30 (32.6)	53 (57.6)
Diploma (13)	6 (46.2)	7 (53.8)	9 (69.2)	9 (69.2)
University (82)	67 (81.7)	43( 52.4)	41 (50.0)	55 (67.1)
	P= 0.000*	P= 0.113	P=0.009*	P=0.010*
Women’s area of specialty				
Medical (7)	6 (85.7)	6 (85.7)	5 (71.4)	7 (100.0)
Admin (19)	16 (84.2)	12 (63.2)	9 (47.4)	11 (57.9)
Profession (23)	13 (65.0)	12 (60.0)	14 (70.0)	15 (75.0)
Academic (21)	38 (77.6)	21 (42.9)	23 (46.9)	31 (63.3)
	P= 0.049*	P=0.013*	P=0.007*	P=0.005*
No. of deliveries				
0 (51)	30 (58.8)	16 (31.4)	21 (41.2)	31 (60.8)
1-3 (124)	89 (71.8)	59 (47.6)	51 (41.1)	70 (56.5)
4-6 (55)	39 (70.9)	9 (52.7)	20 (36.4)	34 (61.8)
7 and more (25)	11 (44.0)	7 (28.0)	7 (20.0)	7 (28.0)
	P=0.029*	P= 0.041*	P= 0.780	P=0.050*

a, Cervical Cancer; *, significant at p˂0.05

Resveratrol (trans-3,5,4´-trihydroxystilbene), a constituent of red wine, has been appeared to have against oxidative properties which is found in excess of 70 types of plants, for example, pines, shelled nut, mulberry and grape(Gusman et al., 2001; Azimi et al., 2015; Azimi et al., 2016). Pathogen contamination, condition changes, daylight, overwhelming metals, and ozone presentation are a few sorts of stresses that incite resveratrol creation in plants (Athar et al., 2007). Previous examinations have demonstrated that the resveratrol has numerous valuable impacts from chemoprevention of tumors to hostile to oxidative, against aggravation, cardio assurance and immunomodulation, in any case the correct atomic component is yet to be cleared up (Dong, 2003). Resveratrol has been shown to be prepared to do avoiding tumor angiogenesis and metastasis and stifles three noteworthy phases of tumor extension including tumor start, tumor advancing, and advancement. The utilization of resveratrol to treat the medication protection for chemoprevention has been archived in light of in vitro and in vivo discoveries (Seve et al., 2005). a few examinations on human cells have demonstrated that different pathways engaged with cell development, cell passing and irritation are managed by resveratrol(Seve et al., 2005). Various examinations from in vitro tries have likewise checked that resveratrol can smother the multiplication of an assorted scope of cells (Aggarwal et al., 2004). Broad examinations have upheld additionally the part of glucocorticoids (GCs, for example, prednisolone in a few cell forms including digestion, separation, and expansion and cell survival. Especially, in lymphoid framework such operators impact the cell cycle, and influence immunoglobulin and cytokine creation, and initiate apoptosis in youthful lymphoblast. GC impacts on invulnerable framework are for the most part required amid the age of the insusceptible collection and in addition the immunomodulation (Schaaf and Cidlowski, 2002; Azimi et al., 2016). In this way these specialists are legitimate contender to be used in the treatment of youth intense lymphoblastic leukemia (ALL) and different malignancies influencing lymphoid framework (Azimi et al., 2016). Among the distinctive instruments by which the malignancy cells may ensure their survival, expansion, and intrusion the MDR-1 has been considered as a key potential giver amid tumor movement. The MDR1 quality encodes the medication transporter P glycoprotein (Pgp) which advances medicate efflux. The most broadly thinks about system by which the human disease what’s more, tumor cell lines oppose to chemotherapy is the over-articulation of Pgp. Different components of medication efflux are intervened by transporter proteins including the multidrug resistance associated protein (MRP) (Zaman et al., 1994) and over-articulation of the significant vault protein (LRP)(Scheffer et al., 1995). Changes in topoisomerase compounds and a modified glutathione/ glutathione -S-transferase (GSH/GST) detoxification framework (Hao et al., 1994) are likewise speak to instruments of cell protection from cytotoxic specialists. 

We have also examined the effect of resveratrol and prednisolone on the gene expression and methylation level of DNA promoters of the MDR1 gene in our previous study and observed that resveratrol and prednisone cause decrease in MDR1 gene expression without altering the methylation level of the MDR1 gene promoter (Zadi Heydarabad et al., 2018).

In another study that was done by Al-Abd et al., (2011)resveratrol inhibited P-gp and reduced the expression of MDR1 in cancer cell lines in vitro. Gautam et al., (2000) illustrated some effects of proliferative inhibition of resveratrol on leukemic cell lines (U937, HL-60) during a series of laboratory studies. 

We have also examined the effect of resveratrol and prednisolone on the gene expression and methylation level of DNA promoters of the MDR1 gene in our previous study and observed that resveratrol and prednisone cause decrease in MDR1 gene expression without altering the methylation level of the MDR1 gene promoter (Zadi Heydarabad et al., 2018).

Due to the low toxicity of resveratrol in vivo as well as in vitro and its anti-leukemic effects, being selective of its effects and be useful as an anti-leukemic agent; We decided to assess the effect of resveratrol and prednisolone on overcoming drug resistance caused by increased expression and function of MDR1 by changing its expression and reducing its expression in CCRF-CEM cancer cells.

## Materials and Methods

*Cell Culture*

As a harmful cell line speaking to ALL, the CCRF_CEM cell line was bought from Pasteur organization of Tehran, Iran. The cells were refined routinely in RPMI 1640 medium supplemented with 10% warmth inactivated fetal cow-like serum (GIBCO, USA), streptomycin (10mg/ml) and 10,000 U/mL of penicillin. The cells were kept up at 37˚C with 5% CO_2_. Preceding resveratrol treatment the cells were seeded in 60mm culture dishes at a thickness of 4 ×10^5^ cells for each dish. 

*Treatment*

Resveratrol (98% immaculateness, Sigma Aldrich, Germany) was broken up in ethanol what’s more, added to culture dishes at the convergences of 15, 50 and 100μM. Following 24 and 48 hours, the cells were gathered for protein extraction. Prednisolone (98 % immaculateness, Sigma-Aldrich, Germany) was broken up in DMSO and added to CCRF-CEM societies at the centralization of 700 μM (Azimi et al., 2015; Azimi et al., 2016) At last, the cells were collected after 24h and 48h for RNA extraction. 

**Figure 1:**
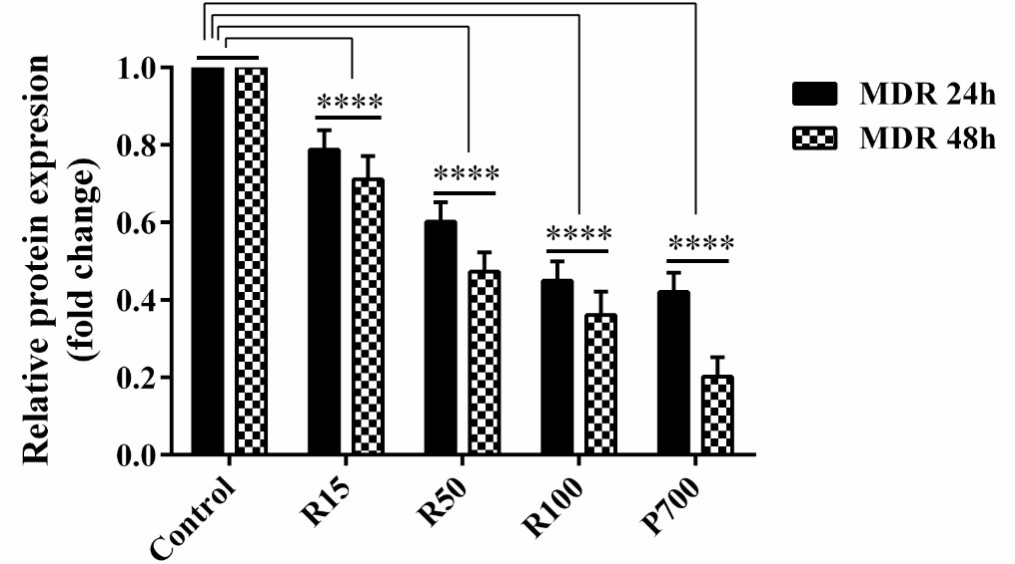
The Effects of Resveratrol and Prednisolone on MDR1 Protein Expression in CCRF-CEM Cells Compared to Control Cells, Cultured with FBS, at 24 and 48 hours after Treatment

**Figure 2:**
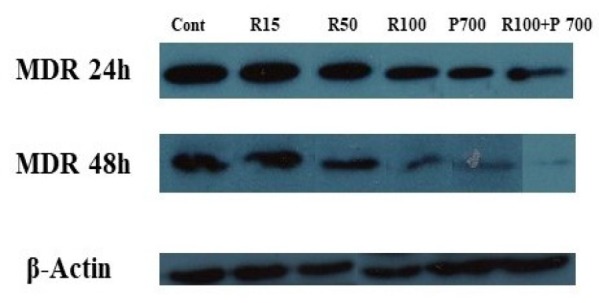
The MDR1 protein level. Results are representative of the reduction in MDR1 at doses of 15, 50 and 100μM of Resveratrol and 700μM of Prednisolone after treatment, as compared to the protein level in control group (untreated cells). The band intensity of MDR1 was quantitated and normalized with b-actin band and expressed as relative MDR1 expression

*Assessment of MDR1 protein*

In order to assess the alteration of protein (MDR1) expression, the Western Blot technique used. Hence, 5×10^6^ cells were cultured in 25cm^2^ flasks in the final volume of 5ml. Subsequently, the cells treated with 15, 50 and 100μM of resveratrol and 700μM of prednisolone for 24 and 48 hours. 

*Extraction and isolation of proteins*

The cells were harvested and washed by cold phosphate buffer saline. Equivalent amounts of cell lysis buffer, including 1% NP-40, 0.5% SDS, 10mM Tris-HCL (pH=7.4), 5mM EDTA, 150mM NaCl, 100µM PMSF, 0.5% sodium deoxycholate, and protease-phosphatase inhibitor were added to cells. 13000RPM was considered to centrifuge samples for 20 min in -4^o^C. Then, the supernatant discarded, and Bradford assay used for evaluation of protein concentration. According to Laemmli assay, the equivalent amounts of protein samples were separated using 10% SDS-PAGE. 

*Western Blot*

Transference of bands from gel to nitrocellulose membrane fulfilled Using Mini Trans-Blot Bio-Rad. After that, the membrane was washed and exposed with blocking solution, containing 5% skim milk in TBS-T, for 1 hour. Subsequently, the cells were washed with TBS-T and exposed to the primary monoclonal antibody in the presence of blocking solution at -4^o^C for a whole night. Anti-actin antibody was considered as an internal control. Then, the membrane was washed in three steps by TBS-T, exposed with secondary antibody, conjugated with HRP, at room temperature for 1 hour. Eventually, chemiluminescence assay was used to the visualization of protein bands.

The effect of resveratrol and prednisolone on CCRF-CEM cells was significant and reduced MDR1 expression. The Western blot method was applied to the measurement semi quantitatively of MDR1 protein expression (Figure 2).

In order to assess changes in MDR1 protein expression, the western blot technique was used. The Actin protein considered as an internal control. Western blot results illustrated decline in MDR1 expression influenced by 15, 50 and 100μM of resveratrol and 700μM of Prednisolone for 24 and 48 hours (Figure 1). As shown in Figure 1 and Figure 2, the treatment of CCRF-CEM cells with 15, 50 and 100μM of resveratrol and 700μM of prednisolone for 24 and 48 hours illustrated a significant decrease of MDR1 expression based on the results obtained from densitometry analysis of MDR1 protein bands. 

Additionally, the western blot technique was used to P-gp (a protein encoded by MDR1) quantitative measurement.

*Statistically Analysis*

In order to do densitometry and assess the bands, the ImageJ software and data analysis carried out by SPSS 20.0 software were used. The immunoblotting and flow cytometry data were obtained from three distinct experiments and demonstrated as a ± mean standard error. For studying the differences between groups, paired T-test was used and the p values < 0.05 were considered as statistically significant.

## Results

*Resveratrol and prednisolone decrease the expression of MDR1 gene without altering the level of methylation of DNA promoters*

In our previous study, to investigate the effect of resveratrol and prednisolone on the level of methylation of MDR1 DNA gene promoters and their effects on MDR1 gene expression in acute lymphoblastic cell CCRF-CEM cells, we measured these cells at doses of 15, 50 and 100 μm of resveratrol and 700 μm of prednisolone and treated them for 24 and 48 hours. Our results in that study showed that resveratrol and prednisone cause decrease in MDR1 gene expression without altering MDR1 gene methylation pattern in a dose and time-dependant manner (Zadi Heydarabad et al., 2018).

*Resveratrol and prednisolone cause decrease in MDR1 protein expression*

As indicated by the past looks into, the convergences of resveratrol required for apoptosis enlistment in the CCRF-CEM cells were 15, 50 and 100μM, while 700μM of prednisolone appeared to be ideal. Likewise, Resveratrol and prednisolone cause a diminishing in MDR1 quality articulation in a period and focus subordinate way. Following 24 and 48 hours of treatment, the varieties of expression levels of MDR1 protein were assessed contrasted and untreated control cells. To look at the adjustments in expression levels of MDR1 protein, the CCRF-CEM cells were treated with resveratrol and prednisolone. It was watched that resveratrol and prednisolone have significant effect on expression levels of MDR1 protein CCRF-CEM cells in the measurements and interims utilized as a part of this investigation. Our result demonstrated that resveratrol in the groupings of 15, 50 and 100μM of Resveratrol and prednisolone 700μM can diminish MDR1 quality articulation (Figure 1 and Figure 2) following 24 and 48 hours of treatment. Having a noteworthy down-direction of MDR1 quality articulation following resveratrol treatment in different diseases (Azimi et al., 2015) we were intrigued to examine the expression levels of MDR1 protein after treatment by resveratrol and in addition prednisolone in various measurements and treatment interims. To play out this, Western blot method was utilized to demonstrate the changes of expression levels of MDR1 protein in these cells. As appeared in Figure 1, we observed changes of expression levels of MDR1 protein following resveratrol and prednisolone treatment taking all things together assigned dosages and interims have Resveratrol and prednisolone cause a diminishing in MDR1 protein expression. 

Moreover, Western blot analysis also showed a significant decrease in MDR1 expression in CCRF-CEM cells treatment with concentration of 15, 50 and 100μM of Resveratrol and 700μM of Prednisolone after 24 and 48 hours (Figure 2). 

Results are representative of the reduction in MDR1 at doses of 15, 50 and 100μM of Resveratrol and 700μM of Prednisolone after treatment, as compared to the protein level in control group (untreated cells). The band intensity of MDR1 was quantitated and normalized with b-actin band and expressed as relative MDR1 expression. 

## Discussion

These days, the chemotherapy, surgery and radiotherapy are the most widely recognized methodologies for treatment of tumor. Considering drug protection, one of the normal strategies to battle malignancies is to sharpen the threatening cells to restorative operators (Gupta et al., 2011; Pouyafar et al., 2018). In spite of the fact that a number of novel obtrusive restorative systems are accessible, a difficult issue in hostile to leukemia treatments is protection from apoptosis which is in any event to some degree a reason of treatment protection. Consequently, apoptosis acceptance is a key component to bring wanted medication proficiency.

Reducing the expression of MDR1 gene can be effective in decreasing the effects of drug resistance, improving the chemotherapy outcome and rescuing patients who are resistant to treatment from the effects of severe toxic chemotherapy (Robert and Jarry, 2003). MDR1 and MRP1 are members of the large ABC family of protein transductors; it is believed that they are an undesirable prognostic factor in the response to and survival of malignancies (Ahani-Nahayati et al., 2018; (Den Boer et al., 1999). In the event of a successful use of natural materials to reduce the effect of drug resistance, we will face some minor side effects. Resveratrol is one of the natural ingredients associated with known anti-cancer effects. These effects associated with the reduction of the expression and function of ABC carriers which reflects a significant reduction in IC50 medications.

Resveratrol is a natural part that has an assortment of organic impacts on malignant cells counting against proliferative, calming and chemo preventive impacts (Cecchinato et al., 2007; Rauf et al., 2018; Zadi Heydarabad et al., 2018). It is cleared up that resveratrol can make the safe harmful cells delicate to treatment through a few systems. It expands the declaration of CD95L and passing receptors interceded apoptosis (Azimi et al., 2015), changes NF-KB pathway and incites customized cell passing(Estrov et al., 2003; Azimi et al., 2016), changes STAT3 articulation by diminishing the capacity of tyrosine kinase Src and directs the survivin acceptance (target quality of STAT3), and fortifies the p53 task and NO generation(Gogada et al., 2011), Besides, GCs act by means of a ligand initiated translation factor known as GC receptor In a wide range of natural examples, the assessment of changes of protein level is a particular procedure for concentrate of expression levels of qualities. In spite of being non-quantitative, western blot is a valuable procedure especially as a confirmation test for prove changes of expression of a protein by two different drugs or substances. 

Considering the above-mentioned items, we investigated whether resveratrol and prednisolone can reduce the expression of MDR1 in the level of protein as a strong barrier against anticancer drugs and one of the main causes of drug resistance, as well as overcome the drug resistance, increasing the expression and function of the MDR1 protein by its reducing (Amin, 2013; Zadi Heydarabad et al., 2018; Ghasemi et al., 2018; Hano et al., 2018). In the present study, our results show that resveratrol and prednisolone, with a dose-dependent effect, can reduce the expression of the MDR1 protein. Reducing the expression of the MDR1 illustrates that resveratrol and prednisolone can overcome the drug resistance by MDR1. In our previous research, we demonstrated that resveratrol and prednisolone can reduce the expression of MDR1 gene without changing the methylation pattern of the MDR1 gene (Zadi Heydarabad, et al., 2018). Our observation on these acute lymphoblastic cells CCRF-CEM also showed that resveratrol and prednisolone can increase the apoptosis of these cancer cells by changing the expression of microRNAs miR 15a and miR16-1 and also reducing the expression of BAX gene as well as increasing expression of BCL-2 without changing the pattern of methylation of the promoter of BAX and BCL-2 genes (Azimi et al., 2015; Azimi et al., 2016; Heydarabad et al., 2016; Zadi Heydarabad et al., 2018; Zadi Heydarabad et al., 2018). In the present study, we also found that resveratrol and prednisolone can overcome drug resistance caused by increasing the expression of MDR1 in acute lymphoblastic cell CCRF-CEM via reducing the expression of MDR1 in the protein level. The issue of overcoming drug resistance has always been an important factor in the elimination of cancer resistant cells to cure. In other studies, such as a study carried out by Quan F, resveratrol reduced the expression of MDR1 (P gp gene) and Bcl 2 (anti-apoptotic) in chemotherapy-resistant epidermoid cell line, KB 200 (Quan et al., 2008). In another study that was done by Al-Abd et al., (2011) resveratrol inhibited P-gp and reduced the expression of MDR1 in cancer cell lines in vitro. Gautam et al., (2000) illustrated some effects of proliferative inhibition of resveratrol on leukemic cell lines (U937, HL-60) during a series of laboratory studies.

Previous studies have shown that resveratrol causes decrease in MDR1 gene expression by inhibiting NF-kB activation as well as the signaling pathway of p38 MAPK (Zhang et al., 2016).

In a study carried out by Vasil F. Chekhun and Galina I. Kulik on the resistance of the human MCF-7 / R breast cancer cell line to doxorubicin, it was concluded that the development of MCF-7 / R drug resistance to doxorubicin, they concluded that the development of MCF-7 / R drug resistance to doxorubicin is the result of a change in the expression of P gp (Chekhun, et al., 2006). Chekhun et al., (2006) reported the relationship between MDR1 promoter methylation and drug resistance in the T-cell line as well as three patients with chronic lymphocytic leukemia. Expression of P-gp is the main barrier in chemotherapy for hematological malignancies, including acute myeloid leukemia (Mahadevan and List, 2004; Chekhun et al., 2006; de Moraes et al., 2013; Gao et al., 2015).

In conclusion, in the present study, ability of resveratrol and prednisolone in reducing the expression of the MDR1, with dose-dependent effect, was observed. Reducing the expression of the MDR1 demonstrates that resveratrol and prednisolone can overcome the drug resistance induced by MDR1.

## Disclosures

The authors state that there is no conflict of interest.
